# Supplementary material for: eDNA metabarcoding as a biomonitoring tool for marine protected areas
Source: PLoS One. 2021 Feb 24;16(2):e0238557. doi: 10.1371/journal.pone.0238557 (PMC7904164; doi:10.1371/journal.pone.0238557)
Supplement: S1 Appendix — (DOCX) [file pone.0238557.s001.docx]

S1 Appendix A – Supplemental Methods

Sample Collection

We conducted our study at Scorpion State Marine Reserve within the Channel Islands National Park and National Marine Sanctuary. To determine the degree to which eDNA could capture documented differences inside and outside this MPA, we sampled three sites: 1) inside the MPA, 2) outside but adjacent (<0.5km) to the MPA (“edge site”), and 3) 2.3k m outside the MPA boundary (“outside site”; Fig 1). At each of these three sites, we sampled directly along a 100 m fixed transect, using a GPS to ensure these transects overlapped with fixed 100 m transects used by the Kelp Forest Monitoring Program for visual monitoring (Kushner, Rassweiler, McLaughlin, & Lafferty, 2013). We collected three replicate 1L water samples from three locations on each transect, totaling 9 spatially structured replicates per site. Due to fieldwork logistical challenges, each site was sampled on a different day with a maximum of 72 hours between sampling events.

We collected seawater samples from 10m below the surface and 1m above the benthos using a 4L Niskin bottle deployed from the UCLA RV Kodiak (Thomsen et al., 2016). From each Niskin deployment, we transferred a single liter of seawater to an enteral feeding pouch (hereafter “pouch”), which we then hung to facilitate gravity filtration through a sterile 0.22 µm Sterivex cartridge filter (MilliporeSigma, Burlington, MA, USA) in the field (Masaki Miya et al., 2016). Additionally, we processed three field blanks as a negative control that consisted of 1L of distilled water following the method above (Goldberg et al., 2016). Finally, we dried eDNA containing Sterivex filters using a 3mL syringe to push through all remaining sea water, and then capped and stored the filters at -20˚C for DNA laboratory work back at UCLA (M. Miya et al., 2015).

**DNA Extraction and Library Preparation**

We extracted eDNA from the Sterivex cartridge using the DNAeasy Tissue and Blood Kit (Qiagen Inc., Germantown, MD) following modifications of Spens et al. (2017), directly adding proteinase K and ATL buffer inside the filter cartridge before an overnight incubation. We PCR amplified the extracted eDNA using the MiFish Universal Teleost *12S* primer (M. Miya et al., 2015) with Nextera modifications following Curd et al. (2019). All PCRs included a negative control where molecular grade water replaced the DNA extraction. For positive controls, we used DNA extractions of Grass carp (*Ctenopharyngodon idella)* and Atlantic salmon (*Salmo salar*), both non-native to California. To ensure amplification success and correct product size, we electrophoresed all PCR products on 2% agarose stained with SybrGreen.

We prepared PCR products for sequencing by pooling 5µL of triplicate PCR reactions (“technical replicates”), cleaning the pooled samples using Serapure magnetic beads (Faircloth & Glenn, 2014) and then quantifying their concentrations using the Quant-iT™ dsDNA Assay Kit (Thermofisher Scientific, Waltham, MA, USA) on a Victor3 plate reader (Perkin Elmer Waltham, MA, USA). We prepared sample DNA libraries using indexes from both the Nextera Index A and D Kit (Illumina, San Diego, CA, UCA) and KAPA HiFi HotStart Ready Mix (Kapa Biosystems, Wilmington, MA, USA) following Curd et al. (2019). We electrophoresed all indexed PCR products on 2% agarose gels to confirm correct product size, and bead cleaned and quantified the resulting libraries as described above. Finally, we pooled Indexed libraries in equimolar concentration, and sequenced the libraries on a MiSeq PE 2x300bp at the Technology Center for Genomics & Bioinformatics (University of California- Los Angeles, CA, USA), using Reagent Kit V3 with 20% PhiX added to all sequencing runs.

**First PCR Protocol**

We performed PCR amplification in triplicate using a 25 μL reaction volume containing 12.5 μL QIAGEN Multiplex Taq PCR 2x Master Mix (Qiagen Inc., Valencia, CA, USA), 6.5 µLof dH2O, 2.5 µL of each primer (2 µmol/L), and 1 μL DNA extraction. PCR thermocycling employed a touchdown profile with an initial denaturation at 95°C for 15 min to activate the DNA polymerase. This was followed by 13 cycles of a denaturation step at 94°C for 30 sec, an annealing step starting at 69.5°C for 30 sec which was then decreased by 1.5°C for each cycle (last cycle was 50°C), and an extension step at 72°C for 1 min. This was followed by 35 additional cycles carried out at an annealing temperature of 50°C using the same denaturation and extension steps above, and ending with a final extension at 72°C for 10 min (Curd et al., 2019).

**Second Indexing PCR Protocol**

This second indexing PCR was performed using a 25 μL reaction mixture containing 12.5 μL of Kapa HiFi Hotstart Ready mix, 0.625 μL of primer i7, 0.625 μL of primer i5, and 10ng of template DNA, and used the following thermocycling parameters: denaturation at 95˚C for 5 min, 5 cycles of denaturation at 98˚C for 20 sec, annealing at 56˚C for 30 sec, extension at 72˚C for 3 min, followed by a final extension at 72˚C for 5 min (Curd et al., 2019).

**Bioinformatics**

To determine community composition, we used the *Anacapa Toolkit* to conduct quality control, amplicon sequence variant (ASV) parsing, and taxonomic assignment using user-generated custom reference databases (Curd et al., 2019). We processed sequences using the default parameters and assigned taxonomy using two reference databases. We first assigned taxonomy using the FishCARD California fish specific reference database (Gold 2020; https://github.com/zjgold/FishCARD). Second, we used the *CRUX*-generated *12S* reference database supplemented with FishCARD reference sequences to assign taxonomy using all available *12S* reference barcodes to identify any non-fish taxa. We transferred the resulting species community tables into *R* for subsequent downstream data analysis (Team, 2014).

**Decontamination**

**Estimation of Index Hopping**

All samples were pooled into a final library and sequenced on a single MiSeq run. Each sample is identified by two sets of molecular barcodes in a unique combination. However, recent evidence has found that there is the potential for indexes to hop from one molecular to another, leading to the incorrect sample assignment during demultiplexing (Costello et al., 2018). To estimate the frequency of index hopping we included a positive control of a non-native fish taxa which we know will not be found in our eDNA samples (Kelly, Gallego, & Jacobs-Palmer, 2018). Index hopping will lead to environmental sequences occurring in the positive control and vice versa. To estimate the frequency of index hopping we modeled the composition of environmental sequences observed on the positive controls and subtract these sequences from the environmental samples run. For example, if 12 reads of Garibaldi (*Hypsypops rubicundus*) are found in the positive control, we subtract 12 reads from the read counts of Garibaldi found in all environmental samples.

**Remove Contamination from Negative Controls**

Here we remove ASVs that occur in positive and negative controls with higher proportions than environmental samples using *R* package *microDecon* (version 1.0.2) (McKnight et al., 2019). We used the standard parameters and grouped samples by location.

**Site Occupancy Modelling**

The goal of site occupancy modeling is to determine whether the presence of an ASV is a true reflection of biological reality (i.e. the fish was present) or the result of a PCR artifact. This is challenging because it requires discriminating between PCR artifacts from rare but real organisms. Site occupancy modeling provides a robust statistical framework to determine if the presence pattern of an ASV reflects a PCR artifact or rare organism (Royle & Link, 2006; Schmidt et al., Kery, Ursenbacher, Hyman, & Collins, 2013). As with all site occupancy models, we assume occupancy state does not change between sampling efforts and that detections at a site are completely independent (Lahoz-Monfort, Guillera-Arroita, & Tingley, 2016). Whether a site is occupied (z=1) or not (z=0) can be evaluated using a Bernoulli trial with probability of occupancy (z ~ Bern(w)). The occupancy probability is constant within a site, which is incorporated in the model through a logit-linear model. The binomial parameter of conditional on site occupancy status was defined as P[i] <- z[i] x P11 + (1-z[i]) x P10 1) where the probability of species occurrences at a location (denoted PSI) the conditional probability of species occurrence within an eDNA sample from a site given that the species was truly at the site (true positive detection) (denoted PS11) the conditional probability of a species occurrence within an eDNA sample from a site given that the species was falsely at the site (false positive detection) (denoted PS10). The probability occurrence function used was the following:

Probability of Occurrence = (PSI x (P11^N) x (1-P11)^(K-N)) / ((PSI x (P11^N) x (1-P11)^(K-N))+(((1-PSI) x (P10^N)) x ((1-P10)^(K-N)))), (1)

Where K is the number of samples taken within a site and N is the number of ASV detections within a site.

Occupancy probability (PSI) was modeled with priors from a left-skewed beta distribution alpha = 1 and beta = 6 as we assume most species are rare in most locations. We modeled the true positive probability (P11) with left-skewed beta distribution alpha = 6 and beta = 1 as we assume our primers to a reasonably good job of detecting species if a species is present. False positive probability (P10) was modeled with priors from a left-skewed beta distribution alpha = 1 and beta = 30 as we assume the false positive rate of detection is unlikely to approach the true positive rate.

For each ASV we created a presence-absence matrix for each site to feed the model. Each matrix was 3 sites by 9 samples. The occurrence of a sequence for one ASV in a given replicate water sample was treated as a detection with that site. Each pattern of occurrence for a given ASV within a given site was considered a case. We then summarized the number of occurrences of each case and ran each case through a separate occupancy model to reduce computational time. Each unique model was run 10 times in order to filter out cases in which the model converged into a local maxima. We then removed all ASVs which had a modeled site occupancy probability of less than 75%.

**Calculating eDNA Index Scores**

The eDNA index was computed following the methods of Kelly et al. 2019 (Kelly, Shelton, & Gallego, 2019). This was accomplished by first calculating the mean read count for each assigned taxonomy and then calculating the relative abundance of each ASV; number of reads of each ASV divided by the total number of reads per sample. The relative abundance of each taxa in each sample was then divided by the maximum abundance for a given species across all samples to generate the eDNA index. The index thus normalizes the read count per species and per sample. The eDNA index values 0 to 1 for each taxa, allowing for abundance comparisons of a specific taxa across sites.

Code for conducting the above decontamination steps is available at https://github.com/zjgold/gruinard_decon .

**Visual Underwater Census Methods**

To assess fish communities using underwater visual census techniques, SCUBA divers from the National Park Service Kelp Forest Monitoring Program followed standard survey protocols following Kushner et al*.* (2013). These protocols include survey types: visual fish transects, roving diver fish counts, and 1m quadrats. The visual fish transects targeted 13 indicator species of fish on visual fish transects recording the counts of adults and juveniles as well as sex for two monandric protogynous hermaphrodite indicator species. This protocol consists of performing 2m x 3m x 50 m transects along the 100m permanent transect. However, during roving diver fish count surveys all positively identified species are recorded. This protocol consists of 3-6 divers counting all fish species observed during a 30 minute time period, covering as much of the 2000 m^2^ of bottom and entire water column as possible (100m long permanent transect, extending 10m both sides, and to the surface). The 1m^2^ quadrat records three small demersal species of fish: *Lythrypynus dalli*, *Rhinogobiops nicholsii,* and *Alloclinus holderi.* All visual surveys occurred along a permanent 100 m transect at each site and were conducted within 2 weeks of eDNA sampling.

**References**

Costello, M., Fleharty, M., Abreu, J., Farjoun, Y., Ferriera, S., Holmes, L., … Mason, T. (2018). Characterization and remediation of sample index swaps by non-redundant dual indexing on massively parallel sequencing platforms. *BMC Genomics*, *19*(1), 332.

Curd, E. E., Gold, Z., Kandlikar, G. S., Gomer, J., Ogden, M., O’Connell, T., … Meyer, R. S. (2019). Anacapa: an environmental DNA toolkit for processing multilocus metabarcode datasets. *Methods in Ecology and Evolution*, *10*, 1469– 1475. doi:https://doi.org/10.1111/2041-210X.13214

Faircloth, B. C., & Glenn, T. C. (2014). Protocol: Preparation of an AMPure XP substitute (AKA Serapure). *DOI*, *10*, J9MW2F26.

Gold, Z. J. (2020). Design and Implementation of Environmental DNA Metabarcoding Methods for Monitoring the Southern California Marine Protected Area Network. UCLA.

Goldberg, C. S., Turner, C. R., Deiner, K., Klymus, K. E., Thomsen, P. F., Murphy, M. A., … Taberlet, P. (2016). Critical considerations for the application of environmental DNA methods to detect aquatic species. doi:10.1111/2041-210X.12595

Kelly, R. P., Gallego, R., & Jacobs-Palmer, E. (2018). The effect of tides on nearshore environmental DNA. *PeerJ*, *6*, e4521.

Kelly, R. P., Shelton, A. O., & Gallego, R. (2019). Understanding PCR processes to Draw Meaningful conclusions from environmental DNA Studies. *Scientific Reports*, *9*(1), 1–14.

Kushner, D. J., Rassweiler, A., McLaughlin, J. P., & Lafferty, K. D. (2013). A multi-decade time series of kelp forest community structure at the California Channel Islands. *Ecology*, *94*(11), 2655.

Lahoz-Monfort, J. J., Guillera-Arroita, G., & Tingley, R. (2016). Statistical approaches to account for false-positive errors in environmental DNA samples. *Molecular Ecology Resources*, *16*(3), 673–685. doi:10.1111/1755-0998.12486

McKnight, D. T., Huerlimann, R., Bower, D. S., Schwarzkopf, L., Alford, R. A., & Zenger, K. R. (2019). microDecon: A highly accurate read‐subtraction tool for the post‐sequencing removal of contamination in metabarcoding studies. *Environmental DNA*.

Miya, M., Sato, Y., Fukunaga, T., Sado, T., Poulsen, J. Y., Sato, K., … Iwasaki, W. (2015). MiFish, a set of universal PCR primers for metabarcoding environmental DNA from fishes: detection of more than 230 subtropical marine species. *Royal Society Open Science*, *2*(7), 150088. doi:10.1098/rsos.150088

Miya, Masaki, Minamoto, T., Yamanaka, H., Oka, S., Sato, K., Yamamoto, S., … Doi, H. (2016). Use of a Filter Cartridge for Filtration of Water Samples and Extraction of Environmental DNA. *Journal of Visualized Experiments*, (117), e54741–e54741. doi:10.3791/54741

Royle, J. A., & Link, W. A. (2006). Generalized site occupancy models allowing for false positive and false negative errors. *Ecology*, *87*(4), 835–841.

Schmidt, B. R., Kery, M., Ursenbacher, S., Hyman, O. J., & Collins, J. P. (2013). Site occupancy models in the analysis of environmental DNA presence/absence surveys: a case study of an emerging amphibian pathogen. *Methods in Ecology and Evolution*, *4*(7), 646–653.

Spens, J., Evans, A. R., Halfmaerten, D., Knudsen, S. W., Sengupta, M. E., Mak, S. S. T., … Hellström, M. (2017). Comparison of capture and storage methods for aqueous macrobial eDNA using an optimized extraction protocol: advantage of enclosed filter. *Methods in Ecology and Evolution*, *8*(5), 635–645. doi:10.1111/2041-210X.12683

Team, R. C. (2014). R: A language and environment for statistical computing. Vienna, Austria: R Foundation for Statistical Computing; 2014.

Thomsen, P. F., Møller, P. R., Sigsgaard, E. E., Knudsen, S. W., Jørgensen, O. A., & Willerslev, E. (2016). Environmental DNA from seawater samples correlate with trawl catches of subarctic, deepwater fishes. *PLoS ONE*, *11*(11). doi:10.1371/journal.pone.0165252
