# Supplementary material for: eDNA metabarcoding as a biomonitoring tool for marine protected areas
Source: PLoS One. 2021 Feb 24;16(2):e0238557. doi: 10.1371/journal.pone.0238557 (PMC7904164; doi:10.1371/journal.pone.0238557)
Supplement: S2 Appendix — (DOCX) [file pone.0238557.s002.docx]

Supplemental Results

There were also significant differences in fish communities when using the Jaccard-Binary similarity distances on the eDNA index scores among the three sites as well as among the three sampling locations along each of the three transects (PERMANOVA p<0.001, betadisper p>0.05). Location along the transect explained 24.0% of the total variance while site (e.g. inside, edge and outside MPA) explained 24.3% of the total variance; 51.7% of the total variance was unexplained. Likewise, NMDS ordination showed weak clustering of samples by both location and site (NMDS, Stress 0.23; S2 Fig). These results are highly concordant to the Bray-Curtis similarity distances on the eDNA index scores suggesting that our findings are robust to the similarity distance metric employed.
