# Supplementary figures and images for: eDNA metabarcoding as a biomonitoring tool for marine protected areas
Source: PLoS One. 2021 Feb 24;16(2):e0238557. doi: 10.1371/journal.pone.0238557 (PMC7904164; doi:10.1371/journal.pone.0238557)

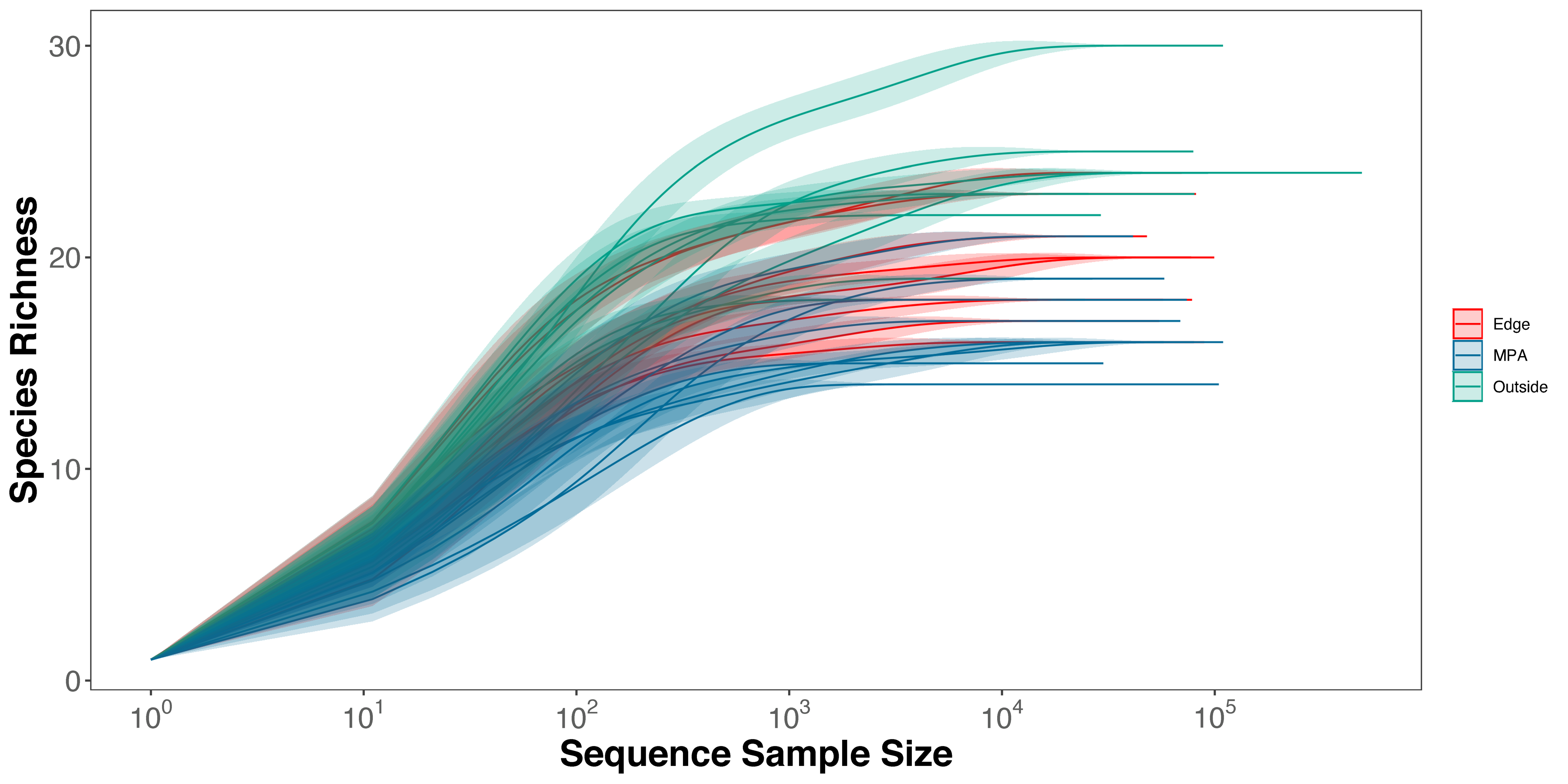

Supplement: S1 Fig — (TIF) [file pone.0238557.s003.tif]

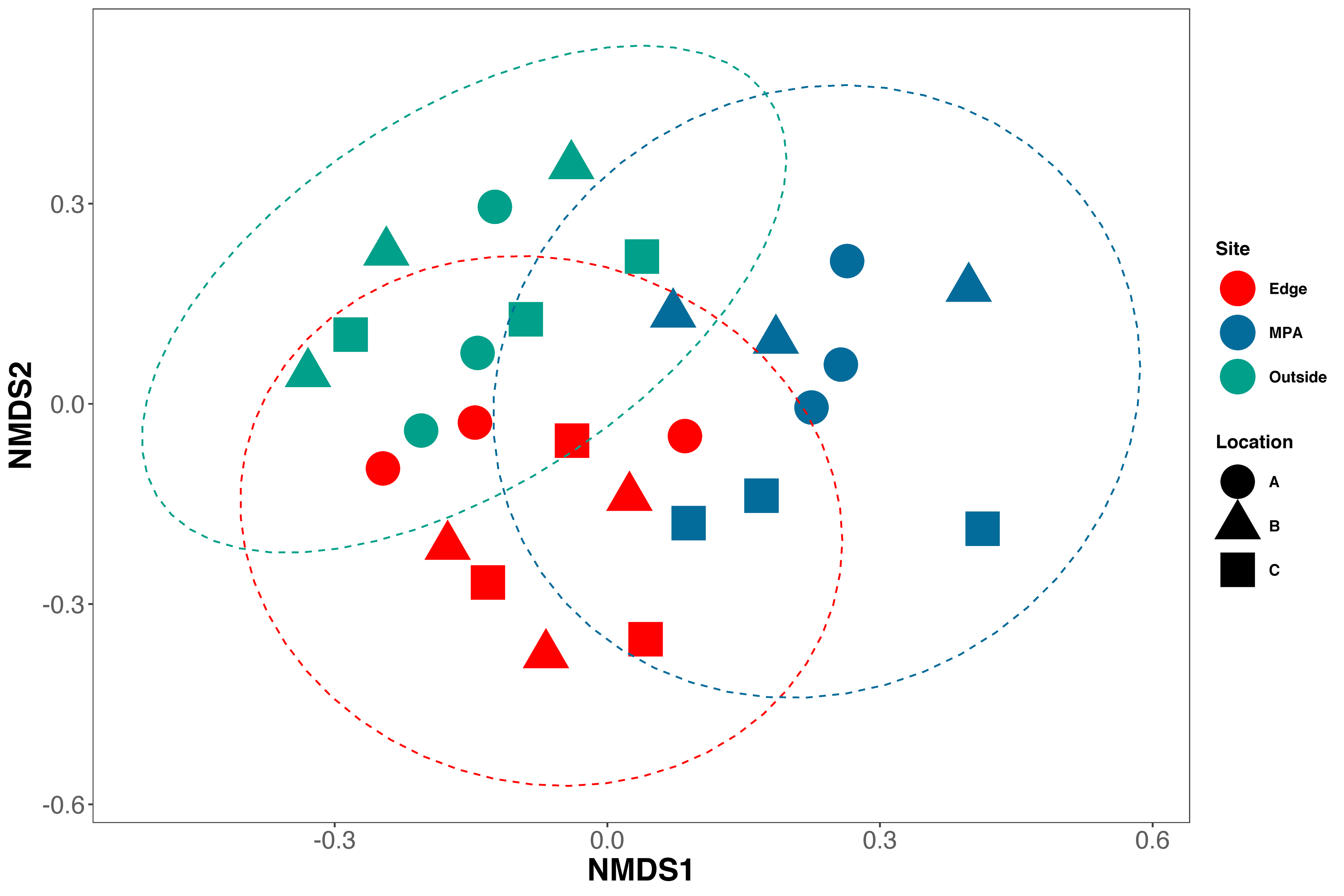

Supplement: S2 Fig — Jaccard-binary dissimilarities were calculated between all samples using only species with occupancy rates over 84%. Samples from Sites (colors) and locations (shapes) are similar to each other (NMDS, Stress = 0.23). (TIF) [file pone.0238557.s004.tif]
